# Recent Developments in Enzyme-Free PANI-Based Electrochemical Nanosensors for Pollutant Detection in Aqueous Environments

**DOI:** 10.3390/polym17101320

**Published:** 2025-05-12

**Authors:** Sarah Cohen, Itamar Chajanovsky, Ran Yosef Suckeveriene

**Affiliations:** 1 Water Industry Engineering Department, Kinneret Academic College on the Sea of Galilee, Zemach 15132, Israel; sarah.cohen00000@gmail.com (S.C.); chk1987@gmail.com (I.C.); 2 Faculty of Engineering, Kinneret Academic College on the Sea of Galilee, Zemach 15132, Israel

**Keywords:** electrochemical nanosensors, conducting polymers, nanomaterials, pollution detection

## Abstract

Wastewater management has a direct impact on the supply of drinking water. New cutting-edge technologies are crucial to the ever-growing demand for tailored solutions for pollutant removal, but these pollutants first need to be detected. Traditional techniques are costly and are no longer competitive in the wastewater cleaning market. One sustainable and economically viable alternative is the fabrication of integrated nanosensors composed of conducting polymers. These include polyaniline doped with various types of nanomaterials such as nanocarbons (carbon nanotubes and graphene), metal oxide nanoparticles/nanostructures, and quantum dots. The synergistic properties of these components can endow sensing materials with enhanced surface reactivity, greater electrocatalytic activity, as well as tunable redox activity and electrical conductivity. This review covers key recent advances in the field of non-enzyme electrochemical conductive polymer nanosensors for pollutant detection in aqueous environments or simulated polluted samples. It provides an introduction to these sensors, their preparation, applications, the environmental and economic hurdles impeding the large-scale development of PANI-based nanomaterials in sensing applications, and future directions for research and real-world applications.

## 1. Introduction

Water, which is crucial for survival, is at the forefront of environmental concerns. Although climate change, geological factors, and natural catastrophes can impact water quality, the intensification of human activity over the last century, mainly due to increased industrialization, urbanization, and farming practices, has played a major role in the decline in water resources [1]. One of the key reasons for the increase in surface water pollution is the release of pollutants into rivers and streams as a result of intensive agriculture and its associated discharge of pesticides, pathogens, and metals [2]. Effluents of wastewater treatment plants, as well as municipal and household activities, also contribute substantially to water pollution since organic and inorganic substances reach rivers and groundwater systems by infiltration into the soil [3].

The main organic contaminants consist of dissolved and undissolved solids such as phenolic compounds, hydrocarbons, and perfluorinated compounds. Today’s prime inorganic contaminants are made up of heavy metals and fertilizers [4]. Polluted water is a danger to both the health of the aquatic ecosystem and to humans. For example, the presence of highly toxic heavy metals such as arsenic, lead, fluoride, cadmium, and mercury can cause respiratory cancer and skin lesions. Exposure to these toxic substances over lengthy periods of time can lead to bladder and lung cancer [5]. According to the World Health Organization (WHO), even low lead concentrations in the blood can impair neurodevelopment in children and have adverse effects on systolic blood pressure in adults [6]. Newer environmental contaminants such as per- and polyfluoroalkyl substances (PFAS) also pose a serious risk to flora and fauna, and by extension to the food chain because of their longevity and bio-accumulative potential [7]. The presence of high concentrations of phenolic compounds in wastewater can cause damage to the heart, kidneys, and liver [8].

Clearly, the assessment, monitoring, and quantification of the concentration of organic and inorganic substances in aqueous environments are crucial for comprehensive water safety programs. Traditional methods based on chromatography and spectroscopy, including atomic absorption spectroscopy, mass spectroscopy, high-performance liquid chromatography and X-ray fluorescence, are costly, time-consuming, and require pre-treatment samples, and can no longer respond to the demand for rapid and high-intensity detection [9,10,11,12,13]. The emerging field of electrochemical nanosensors constitutes one of the most promising alternatives to pollutant sensing that provides high sensitivity and selectivity and achieves very low detection limits [14,15,16].

Nano sensing devices, whose dimensions do not exceed 100 nm, can detect pollutants in trace and at nanoscale [17]. Nanomaterials have unique properties including a high ratio of surface area-to-volume, a superior capacity to identify pollutants at very sensitive levels, a tunable architecture, and sensing properties tailored to detect specific pollutants [18]. These key characteristics give nanosensors multiple advantages, such as high sensitivity and selectivity, low detection limits, miniaturization, real-time sensing, and fast turnarounds of real environmental samples [19].

Electrochemical detection implements advanced functional nanomaterials that enhance electrical conductivity for a specific surface area and with the same mechanical–physical stability as fabricated sensors. Conducting polymers (CPs) and their derivatives are widely used as sensing materials because of their tunable conductivity, easy modification, good environmental stability, and low cost [20]. Polyaniline (PANI) is one of the most widely used [21,22,23]. Its reversible redox properties, i.e., its capacity to switch easily from an oxidized to a reduced state in response to ion transfer, makes it ideal for the development of sensors based on the reception of electrochemical signals [24]. Studies have shown that the functionalization and doping of CPs with different nanomaterials including nanocarbons such as carbon nanotubes and graphene [25,26], metal oxide nanoparticles/nanostructures [27,28,29], and quantum dots [30] result in superior properties that include a larger surface area, enhanced adsorption activity of the targeted pollutant, higher thermal and electrical conductivity, and a high aspect ratio. The incorporation of these nanofillers was found to considerably improve the chemical, physical, and mechanical properties of the synthesized nanocomposites, leading to enhanced sensing performance in terms of sensitivity, selectivity, and accuracy.

**Figure 1:**
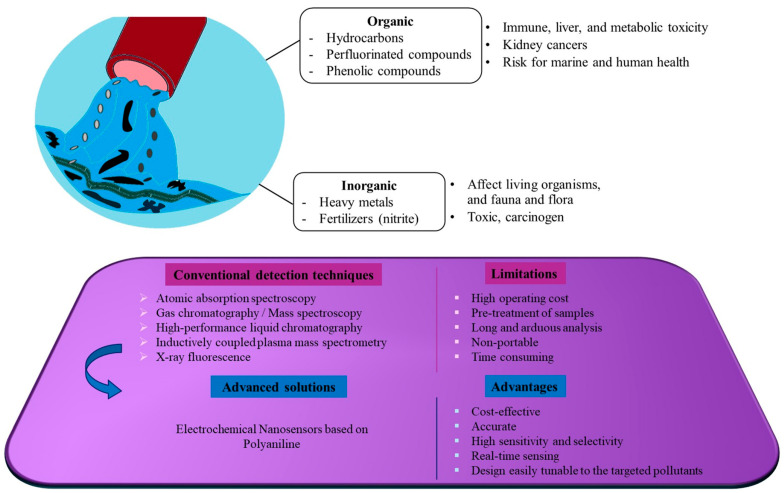
Schematic illustration of hazardous contaminants in water and sensing methods based on PANI described in this review.

This review covers studies examining the performance of enzyme-free PANI-based electrochemical nanosensors designed to sense contaminants in aqueous environments published from 2019 to 2025. Overall, although enzymatic biosensors show remarkable electrochemical characteristics such as high sensitivity and selectivity [31], their inevitable dependency on environmental conditions such as temperature and pH affects their reliability and reproducibility. The high cost of these enzymes and the need to immobilize them on the biosensor surface constitute obstacles to their commercialization [32,33,34]. Figure 1 depicts the major causes of polluted water and the sensing methods based on PANI described in this review to deal with them.

### Search Methodology

For this review, the following databases were searched, Pub Med and Google Scholar for publications in English from 2019 to 2025, using the following search terms: PANI, non enzymatic, electrochemical nanosensor, pollutant sensing and contaminant detection in real water samples. The inclusion criteria were all peer-reviewed articles on the topics corresponding to the search terms. The exclusion criteria were dissertations, non-peer reviewed articles, articles that only provide chemical formulas or discussed techniques with no reported experiments, and articles that conducted contaminant sensing in real non-water samples such as food samples, biological samples, and beverages.

**Figure 2:**
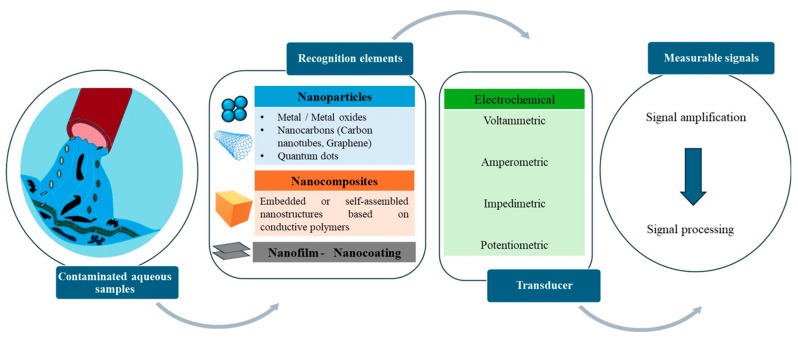
The components of typical electrochemical nanosensors.

This initial search resulted in 150 articles. A careful reading of the abstracts led to the elimination of 100 articles which did not meet the criteria. These articles were then read in full. This led to the exclusion of 15 for the following reasons: the detection limits were not provided and no experiments in real aqueous samples were reported. This left 35 articles which are discussed in this review, as shown in the flowchart below (Figure 2).

## 2. Principles and Preparations of Nanomaterial-Based Nanosensors Using Conducting Materials

### 2.1. How Electrochemical Nanosensors Work

Nanosensors can detect organic and inorganic pollutants in water systems that cause biological, chemical, and physical changes over time. These devices are based on stimulus–response systems that acquire and convert physical, chemical, or mechanical stimuli into a signal that can be recorded and analyzed. When the nanosensor interacts with the targeted analyte, it undergoes a change in its electrical, mechanical, and/or optical properties, which is then converted into a measurable signal by transduction [35]. Nanosensors can be defined by their constituent materials, targeted analytes, and detection mechanisms. Most of the nanosensors for water pollution detection covered in this review rely on electrochemical transduction by voltammetry, amperometry, impedimetric, or potentiometry [36].

Electrochemical sensors consist of a three-electrode system: (i) the working electrode (WE) that is chemically modified when the analytes undergo a redox reaction, (ii) the reference electrode (RE) that maintains a stable potential, and (iii) the counter electrode (CE) [37]. Specifically, the electrochemical sensors evaluate the analyte concentration through chemical reactions. These sensors are made up of a receptor (metal conducive nanoparticles or conductive polymers) and a physicochemical transducer. In dynamic monitoring of the electrochemical response, the receptors form a highly specific interaction with the targeted analyte contaminant through electron transfer. The electrochemical sensing mechanism is based on a redox reaction, i.e., the production or consumption of electrons [2,38,39].

Monitoring via static methods demonstrates the interaction between the receptor and the target analyte is electrostatic since there is no disruption in the electrical signal, and the electrical current is equal to zero [40,41]. The electrical signal generated by the receptor–analyte interaction is then converted into a readable value by a physicochemical transducer. The specific way the contaminant bonds to the sensing polymer causes a change in its conductivity, potential, current, or charge [42].

**Figure 3:**
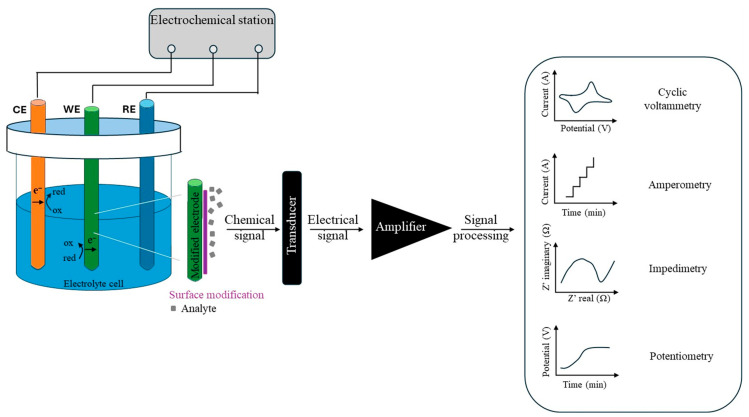
Types of electrochemical sensor mechanisms.

As shown in Figure 3, electrochemical detection methods can be classified according to the type of transduced signal and resulting current waveforms. Voltametric sensors measure the current at different potential points, resulting in a current–voltage curve. They are particularly well suited for sensing trace analytes. Amperometric sensors measure very small currents at constant voltage and yield a current–time curve. Electrochemical impedance spectroscopy sensors are often used to evaluate the concentration of analytes in aqueous solutions. They are used to calculate the impedance of the equivalent electric circuit [43,44]. Potentiometric sensors measure the electrical potential between the working and the reference electrodes at zero current conditions. They are mainly used to detect low analyte ion concentrations in small sample volumes [45,46].

### 2.2. Nanomaterials

#### 2.2.1. Fabrication

The technical prowess involved in the design of miniature, portative, and highly sensitive nanosensors capitalizes on the unique properties of nanomaterials. The term nanomaterials refer to conductive, metallic, and/or carbon-based nanoparticles, nanowires, nanotubes, nanofilms, as well as embedded and self-assembled nanostructures based on polymers and nanoparticles. Nanomaterials are manufactured by bottom-up or top-down methods which enable precise control over the size, morphology, and composition of the nanomaterials. The bottom-up approaches include chemical vapor deposition, sol–gel, self-assembly and spinning. The top-down approaches include lithography, ball milling, and laser ablation [47,48]. To enhance the sensitivity and selectivity of the nanosensor, surface functionalization allows the nanosensor surface to be customized with specific recognition elements that interact with the analyte. Conductive polymers are widely employed in electrochemical sensors because they provide a direct electrical readout in the presence of organic and inorganic pollutants [49].

#### 2.2.2. Conductive Polymers (CPs)

CPs have inherent chemical and physical properties that make it possible to fabricate cost-effective, lightweight, portable, flexible, and tunable nanosensors [49]. CPs can be easily modified chemically with nanoparticles and other polymeric materials. This results in a larger spectrum of tailored nanocomposites. Specifically, functionalization with conductive carbon-based nanoparticles such as nanotubes and graphene considerably enhances the mechanical, optical, and electrical properties of the polymer of interest by providing higher electrical and thermal conductivity, a greater specific surface area, and more mechanical strength [50]. Fayemi et al. [51] reported that the electrochemical properties of carbon quantum dots/PANI-modified screen-printed carbon electrodes produced nanomaterials displaying a larger surface area, higher electrical conductivity, and greater stability than bare electrodes. Copolymerization has also been shown to have considerable advantages, including better processability, polarization, and thermal stability [52]. Most electrochemical nanosensors designed to detect toxic pollutants in water samples are based on polyaniline (PANI), given its high electrical conductivity, high transparency in the visible region, easy functionalization, environmental stability, and low price [53,54,55,56,57]. Desai et al. [58] for example discussed a low cost and non-toxic chronoamperometry sensor based on PANI, graphite, and cobalt ferrite for hydrazine detection in water samples.

## 3. Application of PANI-Based Electrochemical Nanosensors for Pollutant Detection in Aqueous Environments

The intrinsic properties of PANI and its derivates, which include high electrical conductivity, environmental stability, and ease of functionalization, have generated a great deal of attention as electrode modifiers in the field of electrochemical nanosensors [59]. For example, one study examined the cyclic voltammetry (CV) behavior of a PANI-modified glassy carbon electrode (GCE) in an [Fe(CN)6]^2−/3−^ solution and reported enhanced conductivity of the modified electrode [60]. The direct assembly of nano-hybrid materials on the working electrode surface resulted in more active sites, higher stability, perm-selectivity to different pollutants, and strong adherence to the surface electrode [61]. Most pollutants found in water discharge and wastewater consist of (i) heavy metal ions, (ii) phenolic compounds, (iii) organic compounds such as persistent organic pollutants and antibiotics, and (iv) inorganic compounds such as fertilizers. According to the WHO [6], the European Union (EU) [62], and the US Environment protection agency (EPA) [63], the maximum authorized levels for the most hazardous heavy metals in drinking water are (**i**) 3 µg L^−1^ (27 nM, WHO) and 5 µg L^−1^ (44 nM, EU/USA-EPA) for cadmium (Cd);( **ii**) 10 µg L^−1^ (48 nM, WHO/EU/USA-EPA) for lead (Pb); (**iii**) 10 µg L^−1^ (133 nM, WHO/EU/USA-EPA) for arsenic (As); (**iv**) 6 µg L^−1^ (30 nM, WHO), 1 µg L^−1^ (5 nM, EU), and 2 µg L^−1^ (10 nM, USA-EPA) for mercury (Hg); and (**v**) 50 µg L^−1^ (962 nM, WHO/EU) and 100 µg L^−1^ (1924 nM, USA-EPA) for chromium (Cr). These pollutants are discussed in depth in the following sub-sections.

### 3.1. Heavy Metal Ions

Among the advanced materials used to functionalize electrodes, metal–organic frameworks (MOFs) have attracted keen interest as surface modifiers given their unique architecture consisting of metal ions coordinated with multifunctional organic ligands. This new class of porous polymers, when combined with CPs, has a very promising sensing capacity that derives from its synergic properties. These include its high surface area and pore volume that results in durable porosity, good absorption, and excellent catalytic activity in addition to its tunable redox activity and electrical conductivity [64]. Alsafrani et al. [65] fabricated aluminum-succinicate MOF/PANI-modified GCE (Al-SA MOF@PANI/GCE) to electrochemically detect zinc ions in an aqueous solution by linear sweep voltammetry (LSV). The sensor was reported to show good sensing performance within a large linear concentration range of 2.8–228.6 µM with a limit of detection (LOD) and a sensitivity of 0.59 µM and 7.14 µA µM^−1^ cm^−2^, respectively. The detection of zinc ions in real tap water and bottled water samples also exhibited good results in terms of repeatability and recovery, ranging from 96.11% to 98.06%.

**Figure 4:**
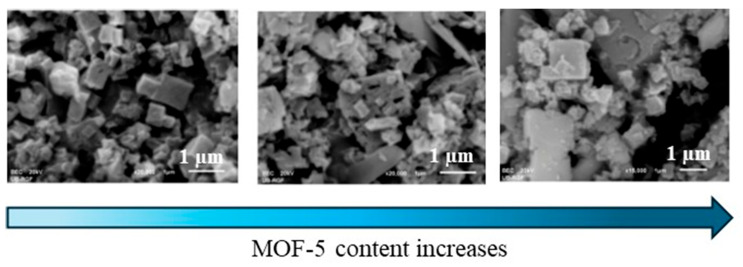
SEM images of MOF-5/PANI-EB at different MOF-5 concentrations [66], adapted from open access journal *Polymers* 2024.

The morphology of MOF/PANI nanocomposites also has a significant influence on the electrochemical and sensing behavior of the resulting sensors. An electrode based on MOF-5, a porous framework composed of [Zn_4_O]^6+^ units connected to linear 1,4-benzenedicarboxylate structural units and PANI in its insulating form (emeraldine base, EB), was designed to detect cadmium (Cd^2+^) and lead (Pb^2+^) ions in water samples using anodic stripping voltammetry (ASV) [66]. The MOF-5/PANI-EB crystalline shape that had the greatest regularity and consisted of cubic particles of submicron size resulted in the highest zeta potential (14.93 mV) and oxidation currents (2.235 mA at −0.68 V and 0.733 mA at −0.46 V for Cd^2+^ and Pb^2+^, respectively) compared to its composite counterparts containing higher MOF-5 content. As seen in Figure 4, the SEM images revealed the presence of irregular shapes with different sized particles as the MOF-5 content increased, due to the formation of larger agglomerates caused by the lower dispersion of MOF-5 within the polymeric matrix. In addition, the MOF-5/PANI-EB-based sensor showed good sensing performance in terms of the simultaneous detection of the two heavy metal ions in both the electrolyte solution and the real water samples.

Nanomaterials comprising noble metals have demonstrated an excellent ability to sense heavy metal ions in aqueous environments in terms of detection limit, sensitivity, and selectivity. Feng et al. [67] used silver (Ag) to boost the electrochemical sensing of arsenic III (As(III)), a very toxic heavy metal. A technique for embedding nanofiber PANI onto the Ag@SiO_2_ core shell was developed (Ag@SiO_2_-PANI NFs). The resulting dendritic three-dimensional network structure (i) prevented Ag@SiO_2_ agglomeration, (ii) enhanced the surface area, and (iii) led to excellent sensing performance in a large linear range of 0.1–100 μg L^−1^ with a LOD of 0.013 μg L^−1^, which is well below the authorized level of arsenic in drinking water.

The conjugation of metallic oxide nanoparticles/nanostructures with PANI also proves their high capacity to adsorb heavy metals in water as a result of their resulting extended surface area. To further improve the binding affinity with heavy metal ions and increase the number of available active sites, other dopants have been incorporated into PANI scaffold-like carbon nanomaterials. For example, a sulfated zirconium oxide/PANI mixture was immobilized on multi-walled carbon nanotube-modified GCE (PANI@ZrO_2_-SO_4_^2−^@MWCNT/GCE) for the detection of Cr(VI) in neutral media. The presence of other ions did not interfere with the Cr(VI) detection except for iron (Fe^3+^) and copper (Cu^2+^) ions [68].

**Figure 5:**
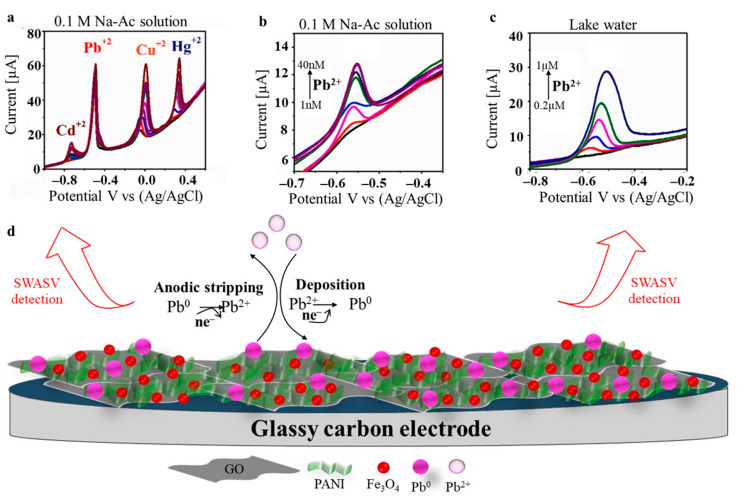
SWASV responses at H-PANI/Fe-GO/GCE leading to (**a**) the simultaneous detection of Cd(II), Pb(II), Cu(II), and Hg(II) at a constant concentration of Pb(II) and increased concentrations of the remaining metal ions from 0.2 to 2 μM (colorful lines); (**b**) the detection of Pb(II) ranging from 1 nM to 40 nM (colorful lines) in 0.1 M Na-Ac solution; (**c**) the detection of Pb(II) ranging from 0.2 µM to 1 µM (colorful lines) in lake water; and (**d**) schematic representation of the adsorption mechanism of Pb(II) at H-PANI/Fe-GO/GCE. Adapted with permission [69]. Copyright Year 2023, Elsevier.

Kumara et al. [69] chemically modified GCE with a hydrogel of PANI doped with Fe_3_O_4_ and graphene oxide (H-PANI/Fe-GO/GCE). They reported excellent sensitivity, stability, repeatability, and reproducibility in the simultaneous square-wave anodic stripping voltammetry (SWASV) detection of the four heavy metals Pb(II), Cd(II), Cu(II), and Hg(II), as seen in Figure 5a. Individual Pb(II) sensing was also tested in both an electrolyte solution (Figure 5b) and in lake water (Figure 5c) where the voltammograms showed similar current peaks. They reported a LOD of 5.15 nM, which is well below the recommended level of lead in drinking water. The Pb^+^ sensing mechanism consisted of three steps: (i) the establishment of strong interactions between the lead ions and the electro-active groups originating from the Fe-GO and PANI, (ii) the reduction of Pb^2+^ to Pb^0^ at a negative potential, resulting in its deposition on the electrode surface, and (iii) anodic stripping of Pb^0^ at a reverse voltage (Figure 5d).

Numerous studies have examined the use of metal-free sensing platforms composed of graphene- and carbon-based materials coupled with PANI for heavy metal ion detection in aqueous media. The synergistic properties stemming from the assembly of these nanocarbon materials with PANI confer multiple advantages on these sensors, including enhanced electrocatalytic activity, a high active surface area, and excellent interactions between the modified electrode and the targeted analytes. For example, a stripping differential pulse voltammetry (SDPV) nanosensor based on brominated white PANI flakes and reduced graphene oxide (rGO) showed a strong capacity to detect Pb(II) and Cd(II) separately and together in various water samples including tap water and industrial wastewater, with a low LOD and a high sensitivity of 7.3 nM–4547.77 µA µM^−1^ cm^−2^ and 6.5 nM–3914.01 µA µM^−1^ cm^−2^, respectively [70].

**Figure 6:**
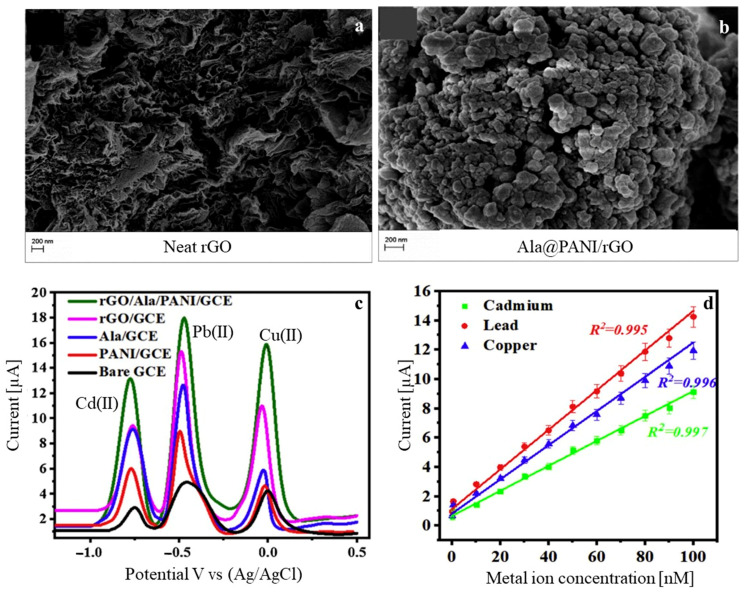
SEM images of (**a**) neat rGO, (**b**) Ala@PANI/rGO, (**c**) SWASV responses for bare GCE and four modified electrodes in an acetate buffer (pH 5) containing 0.2 μM of each metal ion, and (**d**) calibration curves for the three metal ions at Ala@PANI/rGO/GCE. Adapted with permission [71]. Copyright Year 2020, Elsevier.

Other work has also discussed the trace detection of heavy metals. In one study, an alanine@PANI-functionalized rGO (Ala@PANI-rGO) nanocomposite was developed through in situ oxidative polymerization and was analyzed by SWASV [71]. The successful decoration of the rGO sheets with Ala@PANI (SEM images in Figure 6a,b) resulted in a superior sensing capacity conferred by the stronger surface binding affinity after incorporation of the α-amino acids alanine, along with enhanced electrical conductivity and fast electron transfer. Improved electrocatalytic activity was also reported with stripping current signals that were much higher in Ala@PANI-rGO/GCE for the three metal ions, as seen in Figure 6c. High sensitivity and selectivity to Cd(II), Pb(II), and Cu(II) were found, with LOD values of 0.03 nM, 0.045 nM, and 0.063 nM in a broad linear range of 0.08–100 nM, respectively (Figure 6d).

**Figure 7:**
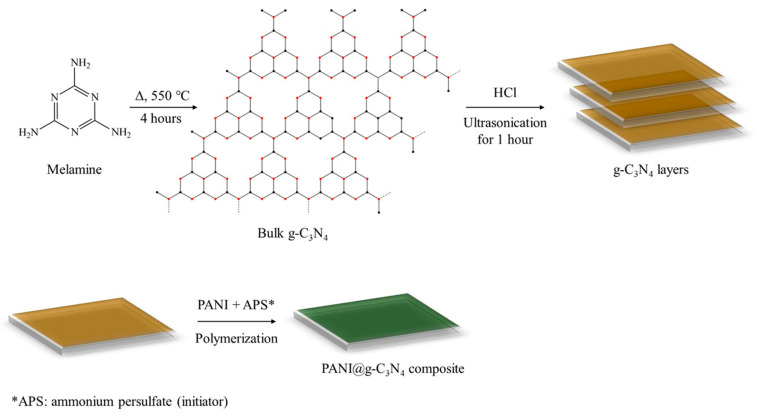
Preparation of PANI@g-C_3_N_4_ composite.

Another study described the modification of GCE with PANI-based hybrid material doped with GO and 3-aminopropyl-triethoxylsilane (PANI@APTES-GO/Nafion/GCE) for lead detection [72]. The material was found to possess good thermal stability, improved electrical conductivity as PANI content increased, and a strong capability to electro-reduce Pb^2+^ to Pb^0^ in both electrolyte solution and real water samples. In another work, a PANI/Graphitic phase carbon nitride composite (PANI@g-C_3_N_4_) was employed to detect cadmium in water using differential pulse anodic stripping voltammetry (DPASV) and showed high electrocatalytic performances including a low detection limit (0.05 µg L^−1^), good reproducibility, stability, and anti-interference capacity [73]. Figure 7 shows the synthesis of the composite resulting in the wrapping of the compact g-C_3_N_4_ by PANI.

The adsorption mechanisms of Pb(II) and Hg(II) at the surface of PANI/Quinoxaline-modified GCE (PANI@QUA/GCE) have been studied by applying density functional theory (DFT) and showed that lower HOMO-LUMO energy gaps were obtained for PANI@QUA/GCE compared to PANI/GCE and QUA/GCE in addition to higher electron affinity and electronegativity when sensing the two heavy metal ions [74].

PANI can also be fabricated via molecular/ion imprinting techniques that give unique physicochemical properties to electrode materials. This powerful molecular or ion imprinted polymer (MIP or IIP) technique is based on the creation of artificial recognition sites in a polymeric matrix that can attract a specific analyte (molecule or ion) in terms of shape or size. The template analyte is first complexed with the functional monomer prior to polymerization and is then extracted, which leaves imprinted cavities in the matrix [75]. For example, an IIP bismuth modified-carbon paste electrode (CPE-Bi) was developed by the electropolymerization of aniline as the functional monomer and nickel sulfate as the source of Ni(II) ions to create Ni(II) templates (IIPANI/CPE-Bi) [76]. The authors posited that the pair of free electrons of aniline would interact with Ni(II). The Ni-templated imprints yielded high sensitivity and selectivity to Ni(II) in real water samples, with a LOD of 4.82 nM. Wu et al. [77] developed an imprinted PANI/gold nanoparticle (PANI-AuNPs) nanocomposite sensor. This modified GCE exhibited high sensitivity and selectivity and good anti-interference ability in Cd(II) detection in water samples.

### 3.2. Phenolic Compounds

**Figure 8:**
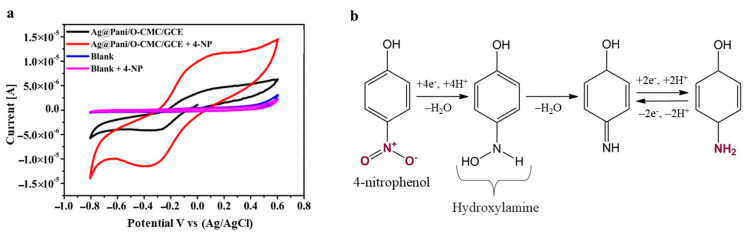
(**a**) CV curves for bare GCE (blank) and Ag@PANI/O-CMC/GCE in 0.10 M PBS (pH = 6.0) with and without 0.10 mM 4-NP at 0.1 V s^−1^ [79] and (**b**) 4-NP reduction mechanism [80]. Adapted with permission [79]. Copyright 2025, Elsevier. Adapted with permission [80]. Copyright 2020, Elsevier.

The other major contaminants of aquatic media are phenolic substances. An electrochemical pH-based sensor was fabricated to detect aminophenol (AP) traces in an aqueous solution using electrospun microfibers of PANI/rGO reinforced with polycaprolactone (PCL) known as F-PANI/rGO/PCL [78]. The resistivity response was monitored as a function of the analyte concentration. The sensor showed high electrical conductivity, sensitivity, and selectivity, and an ultra-low LOD of 8.34 nM, primarily due to the morphology of the nanocomposite, i.e., stretched microfibers with a highly interconnected web-like pattern that resulted in the high dispersion of rGO within the polymeric matrix. Al-Ghamdi et al. [79] discussed 4-nitrophenol (4-NP) sensing in both an electrolyte solution and real samples using an Ag-anchored oxidized-carboxymethyl cellulose embedded PANI-modified electrode GCE (Ag@PANI/O-CMC/GCE). The LOD and sensitivity were reported to be 0.58 nM and 2.48 µA nM^−1^ cm^−2^ in a broad dynamic range of 10–100 nM, respectively. The CV curves for the nanocomposite-modified electrode in 0.10 M PBS (pH = 6.0) in the absence and presence of 0.10 mM 4-NP (Figure 8a) showed a stronger reduction at peak current and a higher current value upon the addition of 4-NP, hence confirming that the rapid electron transfer enabled catalysis of the electro-reduction of 4-NP. In tap water, the recoveries were between 99.9% and 101%, thus showing good sensitivity. A PANI-decorating GO-iron tungsten nitride nanocomposite (PANI-GITN) was also developed to detect 4-NP in aqueous media [80]. This sensor exhibited excellent performance, with a LOD and sensitivity of 5.2 nM/253.08 μA μM cm^−2^ and 2.4 nM/354.92 μA μM cm^−2^ for the oxidation and reduction peaks, respectively. The 4-NP mechanism consisted of the reduction in the nitro group into the corresponding amino group via hydroxylamine, as shown in Figure 8b. Its high performance was attributed to (i) the planar nanostructure after GITN incorporation that made more binding sites available, (ii) PANI’s high electrical conductivity, (iii) rapid electron transfer by GITN, and the GO that can form unique interactions with 4-NP via hydrogen bonds, π-π stacking, and electrostatic, and (iv) low interfacial charge transfer resistance. The 4-NP detection in different types of real aqueous media also showed very high recoveries ranging from 94.2% to 108%.

Simultaneous DVP detection of benzenediol isomers (hydroquinone, HQ; catechol, CC; resorcinol, RS) was successfully achieved in an acidic medium using a Prussian blue-doped nanosized PANI nanocomposite prepared by an in situ mechanochemical reaction between aniline and iron(III) chloride salt that resulted in nanosized PANI emeraldine salt, which was subsequently doped with K_4_Fe(CN)_6_ given Pb@NS-PANI [81]. The redox HQ to CC reaction involved the transfer of two electrons, yielding p-quinone and O-quinone, respectively, unlike RS, which underwent a keto-enol tautomerism reaction via the transfer of one electron, thus leading to the formation of three different RS radicals. The LODs were reported to be 180 nM, 10 nM, and 20 nM for HQ, CC, and RS, respectively. However, better sensitivities to HQ and CC were obtained by utilizing cobalt-doped tin oxide (Co@SnO_2_) with PANI (Co@SnO_2_-PANI) in an alkaline medium, i.e., 4.94 nM and 1.5786 nM, respectively [82]. The remarkable PANI conductivity enhanced the catalytic activity of Co@SnO_2_, thus enabling the linear response sensor to provide outstanding levels of sensitivity of 2 × 10^−2^ to 2 × 10^−1^ M.

### 3.3. Organic Compounds

The other main category of pollutants present in water consists of organic residues. A formaldehyde sensor was developed by modifying the working electrode with PANI@CuO. CuO (metal oxide quantum dots) possesses a specific affinity with formaldehyde which makes it possible to catalyze the oxidation reaction that was reported to yield an accurate, reliable sensor for 720 h [83].

**Figure 9:**
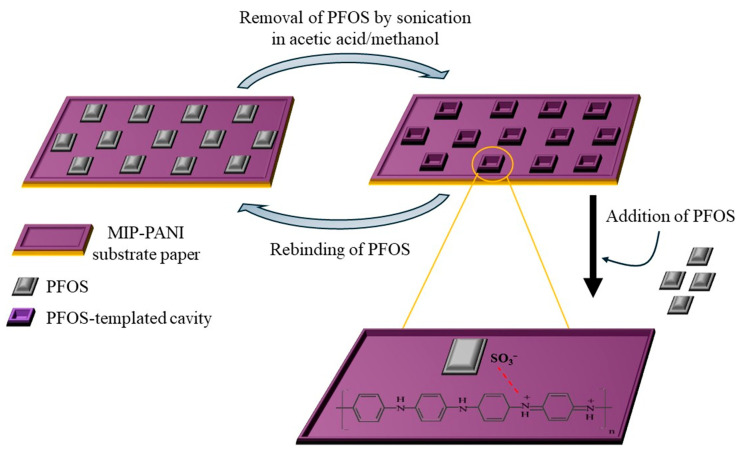
Schematic representation of removal/rebinding MIP mechanism of PFOS on MIP-PANI-based paper substrate.

Persistent organic pollutants (POPs) are hazardous chemicals and a known threat to human health and marine ecosystems because of their longevity and accumulation in the environment. Their derivates, such as perfluorooctanoic acid (PFOA), are toxic chemicals as well that can enter the food chain and drinking water. PFOA was successfully detected by a DVP sensor based on PANI/Chitosan at a 1:1 ratio (PANI@CHT) and achieved a sensitivity of 1.08 ppb in the large linear range of 5–150 ppb [84]. In another study, the perfluorooctanesulfonic acid (PFOS) concentration, another toxic persistent pollutant, was monitored in water samples by utilizing MIP-PANI on a paper substrate [85]. The PFOS-templated cavities were fabricated by sonicating the MIP-PANI paper for 4 h in a cosolvent of acetic acid/methanol (Figure 9). The change in resistivity of the fabricated electrode was monitored as a function of the PFOS concentrations. A good LOD of 1.02 ppt was achieved.

**Figure 10:**
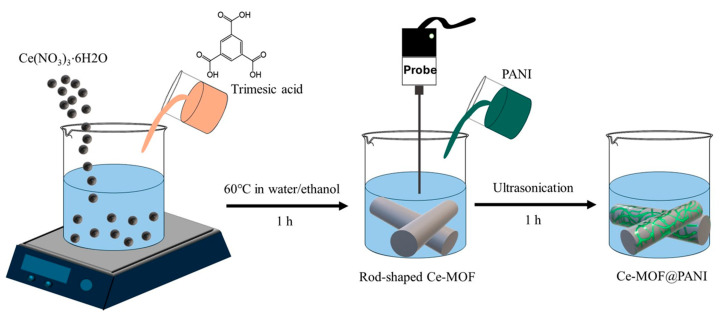
A schematic illustration of the preparation of the Ce-MOF@PANI nanocomposite.

Similarly to POPs, the overuse of organochlorine pesticides can lead to their persistence in aqueous environments. Their non-biodegradability makes them extremely toxic to animals and humans. A number of studies have reported on non-enzymatic sensing in aqueous media. For example, Masibi et al. [86] reported on the square-wave voltammetry (SWV) detection of endosulfan, a widely used pesticide in agriculture, which involved utilizing a nanocomposite based on PANI, antimony oxide nanoparticles, and single-walled CNTs (AONP@PANI-SWCNT). The sensor displayed a linear response of 32.3–77.6 µM with a detection limit of 6.8 µM and a sensitivity of 0.2086 µA µM^−1^. The sensing of 2,4-dichlorophenoxyacetic acid (2,4-D), a systemic herbicide that is acknowledged to be a toxic pollutant and carcinogen, consisted of using an Fe_3_O_4_-PANI nanocomposite prepared by chemical oxidative polymerization of an aniline in acidic medium in the presence of magnetic nanoparticles and APS [87]. A low resistance value as well as a high sensitivity of 4.62 × 10^−7^ μA μM^−1^ cm^−2^ with a detection limit of 0.21 µM were obtained via the synergism between Fe_3_O_4_ and PANI that led to improved nanocomposite conductivity, a larger surface area, and a higher number of adsorption sites. Zhou et al. [88] investigated the detection of carbendazim (CBZ), a toxic pesticide, using a Cerium-MOF@PANI (Ce-MOF@PANI) nanocomposite dripped on carbon cloth (CC) in a neutral medium. Figure 10 depicts the preparation of Ce-MOF@PANI. They reported a higher electrochemical response and enhanced catalytic oxidation of CBZ compared to the pristine Ce-MOF-based electrode as a result of the π-π stacking between CBZ and the nanocomposite, coupled with the nanosized pores of Ce-MOF@PANI that facilitated the accumulation of CBZ at the electrode surface.

Other types of enzyme-free sensors have been developed to detect organic pollutants in water such as antibiotics. An amperometric amoxicillin (AX) sensor was prepared without the presence of enzymes by modifying the screen-printed carbon electrode (SPCE) with a functionalized PANI-silver bromide (PANI-AgBr) composite [89]. This sensor exhibited excellent selectivity, repeatability, and stability with a very low LOD of 0.193 nM in a linear range of 0.193–0.855 nM. A nanocomposite based on PANI and TFAB-COF, a covalent organic framework prepared by Schiff-based condensation of 2,4,6-tris-(4-formylphenoxy)-1,3,5-triazine and p-aminobenzoyl hydrazide, was used to sense sulfamethoxazole (SMX) in an aqueous environment [90]. This sensor was found to be highly sensitive, accurate, stable, and interference-resistant when monitoring different SMX concentrations in real tap water and Yellow River water samples. Chuiprasert et al. [91] reported the electrochemical detection of ciprofloxacin (CIP) using an electropolymerized PANI and poly(*o*-phenylenediamine) (*o*-PDA)-MIP, which was then coated on rGO/GCE (PANI−*o*-PDA MIP@rGO/GCE). The DVP sensor displayed high sensitivity and selectivity with a detection limit of 0.00005 μmol L^−1^ in a large linear range of 0.001−0.5 μmol L^−1^.

### 3.4. Inorganic Compounds

Nitrite is now classified as a carcinogenic inorganic fertilizer. This has led to a reduction in its utilization in agriculture and industry. In drinking water, the admissible nitrite concentration is below 3 mg L^−1^ (65.21 µM) [6]. Several studies have discussed its detection. For example, PANI was ultrasonically polymerized in the presence of manganese dioxide that acted as an oxidant, and the resulting sensor showed a good detection limit of 1.08 µM, an enhanced adsorption of nitrite via the presence of functional amine and imino PANI groups, and recoveries exceeding 95% in real water samples [92]. Patri et al. [93] modified GCE with PANI, carbon nanofibers, and zeolite imidazole frameworks. The modified electrode ZIF-8/CNF/PANI/GCE exhibited a good detection limit of 8.1 µM in broad linear nitrite concentrations of 16 to 835 µM as well as good interference resistance even in the presence of biomolecules such as glucose and dopamine. By utilizing copper nanoparticles coated with an antioxidant film based on PANI and carboxymethylcellulose sodium, the modified electrode PANI-CMC@CuNPs/GCE showed higher sensitivity at 0.170 µM in addition to good stability 25 days later [94]. The linear amperometric response sensor, which was prepared by casting a layer of PANI on GCE followed by casting a layer of nickel oxide nanoflowers (NiOnf), provided excellent LODs of 0.064 µM and 0.0097 µM at high (1–500 µM) and low (0.1–1 µM) concentrations, respectively [95]. This was attributed to the highly porous structure of NiOnf that creates the larger surface area, associated with the specific intermolecular interactions between the nitrite ions and PANI.

The sensing of other inorganic contaminants in water such as phosphate [96] and ammonium ions [97] have been studied. Table 1 summarizes the sensing performances discussed in this section.

## 4. Conclusions and Future Directions

Overall, this review shows that electrochemical nanosensors based on PANI can detect diverse organic and inorganic compounds in aqueous environments without the use of enzymes. Most CPs exploit the heightened electrocatalytic activity of PANI to produce conducting polymer-based nanostructures that demonstrate higher electrical conductivity, a larger surface area, and a better electron transfer rate. The assembly of PANI with other nanomaterials, including carbon nanotubes, graphene and its derivatives, as well as metal and metal oxide nanoparticles/nanostructures considerably improves the adsorption of the targeted analytes at the electrode surface as a result of the larger specific surface area, more specific recognition sites, and greater binding affinity. The synergistic properties of these conjugated conducting polymers show better sensitivity, selectivity, and stability to sensing contaminants in water samples. Molecular/ion imprinted polymerization thus emerges as a very powerful technique for the detection of heavy metal ions and small molecules. The formation of specific cavities arising from the incorporation of the analyte template followed by its removal from the polymeric matrix endows the resulting sensors with greater sensitivity, selectivity, and accuracy.

Nevertheless, as noted by Kaur et al. [98], the fabrication of nanomaterials must overcome several hurdles before more comprehensive water supply programs can be developed. On the one hand, the high cost of nanoparticles (NPs) currently prevents large-scale industrial applications, which impacts the future development of nanotechnology. The commercialization of PANI-based sensors still remains a challenge as well because of the difficulty of achieving the uniform dispersion of nanofillers within the polymeric matrix. Although a multitude of publications have reported the successful fabrication of highly sensitive, accurate, portable, and real-time detection apparatuses, most never move beyond the prototype stage. Closer collaboration between engineering and the sciences could help commercialize these sensors. For example, a relative cost estimate study comparing a zinc/iron layered double hydroxide/PANI-modified carbon paste electrode with two conventional detection techniques, i.e., inductively coupled plasma (ICP) atomic spectrometry and atomic absorption spectrometry (AAS), for heavy metal detection [99] showed that the overall cost was estimated as USD 1.25, which represents less than 0.1% of the total cost required to conduct the same adsorption study using ICP or AAS. Hence, the use of a disposable electrode based on PANI nanomaterials is truly cost-effective. Future work should also explore the toxicity of NPs to assess the impact of long-term exposure and possible adverse environmental effects.

## Figures and Tables

**Table 1:** PANI-based electrochemical nanosensors.

Technique	Analyte	Electrochemical Platform	LOD [nM]	Sensitivity [µA µM^−1^ cm^−2^]	Linear Range [µM]	Ref
CV	–	PANI NPs/GCE	–	–	60–180	[60]
LSV	Zn(II)	Al-SA MOF@PANI/GCE	590	7.14	2.8–228.6	[65]
ASV	Cd(II) Pb(II)	MOF-5/PANI-EB	103 100	156 27 [AM^−1^]	0.7–1.5 0.7–1.2	[66]
SWASV	As(III)	Ag@SiO_2_-PANI NFs/SPCE	0.013 [μg L^−1^]	0.83 [μA μg^−1^ L]	0.1–100 [μg]	[67]
DVP	Cr(VI)	PANI@ZrO_2_-SO_4_^2−^@MWCNT/GCE	64.3	1.3693 0.9832 [μA Lμmol^−1^]	0.55–13.7, 13.7–39.5	[68]
SWASV	Pb(II)	H-PANI/Fe-GO/GCE	5.15	0.0003304	1–40 (×10^−3^)	[69]
SDPV	Cd(II) Pb(II)	rGO-Brominated White PANi Flakes/GCE	6.5 7.3	3914.01 4547.77	0.01–0.23 0.01–0.18	[70]
SWASV	Cd(II) Pb(II) Cu(II)	Ala@PANI-rGO/GCE	0.03 0.045 0.063	0.00043 0.00061 0.00071	0.08–100 (×10^−3^)	[71]
LSV	Pb(II)	PANI@APTES-GO/Nafion/GCE	5.3	165.71 [µA µM^−1^ cm^−1^]	0.01–0.4	[72]
DPASV	Cd(II)	PANI@g-C_3_N_4_/GCE	0.05 [μg L^−1^]	–	0.1–140 [μg L^−1^]	[73]
DVP	Hg(II) Pb(II)	PANI@QUA/GCE	1.28 5.91	–	1–20	[74]
DPSAV	Ni(II)	IIPANI/CPE-Bi	4.82	–	0.01–1	[76]
SWASV	Cd(II)	PANI-AuNPs/GCE	1.2 [μg L^−1^]	–	5–100	[77]
pH sensor	AP	F-PANI/rGO/PCL	8.34	123.8 [kΩ µM^−1^]	0.061–0.31	[78]
DVP	4-NP	Ag@PANI/O-CMC/GCE	0.58	0.00248	0.01–0.1	[79]
CV	4-NP	PANI-GITN/GCE (oxidation/reduction)	5.2 2.4	253.08 354.92	0.03–3 0.01–4	[80]
DVP	HQ CH RS	Pb@NS-PANI/GPE	180 10 20	–	1–350.5 2–350.5	[81]
DVP	HQ CH	Co@SnO_2_–PANI/GCE	4.94 1.5786	9.68 12.80 [µA cm^−2^]	2 × 10^−2^–2 × 10^−1^ M	[82]
Amperometric	FA	PANI@CuO	10^−6^ [mol/L]	–	–	[83]
DVP	PFOA	PANI@CHT/SPCE	1.08 [μg L^−1^]	–	5–150 [μg L^−1^]	[84]
pH sensor	PFOS	MIP-PANI	1.02 [ppt]	–	1–100 [ppt]	[85]
SWV	EDS	AONP@PANI-SWCNT/GCE	6800	0.2086 [µA µM^−1^]	32.3–77.6	[86]
Amperometric	2,4-D	Fe_3_O_4_-PANI/GCE	210	4.62 × 10^−7^	1.35–2.7	[87]
DVP	CBZ	Ce-MOF@PANI/CC	12.6	–	0.1–80	[88]
Amperometric	AX	PANI-AgBr/SPCE	0.193	0.04	0.193–0.855 (×10^−3^)	[89]
DVP	SMX	TFAB-COF@PANI/GCE	107	–	1–450	[90]
DVP	CIP	PANI−*o*-PDA MIP@rGO/GCE	0.05	5.78 × 10^6^ [μA μmol^−1^ L]	0.001−0.5	[91]
CA	Nitrite	PANI@MnO_2_/GCE	1080	0.225	19.98–732.17	[92]
SWV	Nitrite	ZIF-8/CNF/PANI/GCE	8100	–	16–835	[93]
Amperometric	Nitrite	PANI-CMC@CuNPs/GCE	170	0.113 0.049	3–15,000 15,000–29,000	[94]
Amperometric	Nitrite	PANI/NiOnf/GCE	9.7 64	–	0.1–1 1–500	[95]
CA	Phosphate	AHM@PANI/CC/PVDF	600	0.0648 [µA µM^−1^]	10–114	[96]
CA	NH4^+^	PANIep@Au/GCE	30 70	0.34 [mA/µM] 0.18 [mA/µM]	0.35–1.5 2–7	[97]

**CV**: cyclic voltammetry; **LSV**: linear sweep voltammetry, **ASV**: anodic stripping voltammetry; **SWASV**: square-wave anodic stripping voltammetry; **DVP**: differential pulse voltammetry; **SDPV**: stripping differential pulse voltammetry; **DPASV**: differential pulse anodic stripping voltammetry; **SWV**: square-wave voltammetry; **CA**: Chronoamperometric; **AP**: aminophenol; **4-NP**: 4-nitrophenol; **HQ**: hydroquinone; **CH**: catechol; **RS**: resorcinol; **FA**: formaldehyde; **PFOA**: perfluorooctanoic acid; **EDS**: endosulfan; **2,4-D**: 2,4-dichlorophenoxyacetic acid; **CBZ**: carbendazim; **AX**: amoxicillin; **SMX**: sulfamethoxazole; **CIP**: ciprofloxacin; **GCE**: glassy-carbon electrode; **CPE**: carbon paste electrode; **SPCE**: screen-printed carbon electrode; **GPE**: graphite paste electrode; **CC**: carbon cloth.
